# The metabolic trap: Candida parapsilosis inhibits Staphylococcus aureus biofilm maturation by disrupting pH homeostasis and inducing premature exodus

**DOI:** 10.1099/jmm.0.002186

**Published:** 2026-07-28

**Authors:** Ciara Furlong, Salini Mohapatra, Denise Harold, Emma Finlay, Linda Holland

**Affiliations:** 1 School of Biotechnology, Dublin City University, Dublin 9, Ireland

**Keywords:** biofilm dispersion, *Candida parapsilosis*, metabolic trap, nuclease, polymicrobial biofilm, *Staphylococcus aureus*

## Abstract

**Introduction.** Hospital-acquired infections (HAIs) frequently manifest as device-related biofilms that exhibit enhanced tolerance to conventional therapies contributing to antimicrobial resistance. Polymicrobial biofilms involving *Candida* and *Staphylococcus* species are a major cause of persistent nosocomial infections. However, while the synergism between *Candida albicans* and *Staphylococcus aureus* is well-characterized, the interactions involving non-albicans *Candida* remain poorly understood.

**Hypothesis/Gap Statement.** The specific interactions between *Candida parapsilosis* and *S. aureus* were entirely unknown, although it was broadly assumed they would be synergistic in nature, mirroring known *Candida–Staphylococcus* models.

**Aim.** This study investigated the interspecies dynamics between *C. parapsilosis* and *S. aureus* within a mixed biofilm context.

**Methodology.** *C. parapsilosis* secretome fractions were isolated and screened against methicillin-sensitive (MSSA) and methicillin-resistant (MRSA) *S. aureus* strains. Their effects on biofilm formation, primary attachment, planktonic growth and eradication were evaluated under varying glucose concentrations, followed by transcriptomic analysis of treated staphylococcal cells.

**Results.** We report the discovery of a small (<10 kDa), heat-stable fungal-secreted factor that significantly inhibits the maturation of MSSA biofilms and disperses preformed biomass without affecting primary attachment or planktonic growth, although MRSA strains remained recalcitrant. This antagonism is strictly glucose-dependent; the inhibitory effect is potent in 0.2% glucose but is abolished in both 0.5 and 1.0% glucose. Transcriptome analysis revealed that the fungal secretome triggers a pleiotropic ‘Metabolic Trap’ in *S. aureus*, characterized by the downregulation of the glycolytic pathway (e.g. *tpiA*, *gapA*) and a failure to induce critical-acid-tolerance systems, including the arginine deiminase and urease operons. This metabolic reprogramming maintains a near-neutral local pH (5.8–6), which in turn provides an optimal environment for the observed upregulation of staphylococcal nuclease (*nuc*) ultimately degrading the extracellular matrix and preventing the development of a mature biofilm architecture.

**Conclusion.** We propose that the *C. parapsilosis* secretome effectively tricks *S. aureus* into a premature exodus phase, where nuclease-mediated matrix degradation prevents the establishment of a stable biofilm architecture. These findings underscore the highly species-specific nature of fungal–bacterial interactions and identify a specific metabolic vulnerability in *S. aureus* that may be exploited to develop novel anti-biofilm strategies against polymicrobial communities.

## Data Summary

The transcriptomic datasets generated and analysed during the current study are available in the National Center for Biotechnology Information (NCBI) Gene Expression Omnibus repository under accession no. GSE334781. The analytical pipelines, statistical criteria and software parameters used to process these data are fully detailed in the Methods section.

## Introduction

Hospital-acquired infections (HAIs) remain a critical burden on global public health, frequently manifesting as device-related infections such as central line-associated bloodstream infections (BSIs), catheter-associated urinary tract infections and ventilator-associated pneumonia [1]. A primary driver of HAI persistence is the formation of biofilms which are structured microbial communities encased in a self-produced extracellular matrix [2]. The National Institutes of Health estimates that biofilms are associated with over 80% of all human microbial infections [3, 4]. Within these niches, micro-organisms exhibit significantly enhanced tolerance to both the host immune response and conventional antimicrobial therapies, directly contributing to the rising global challenge of antimicrobial resistance (AMR) [5].

Clinical biofilms are increasingly recognized as complex, polymicrobial ecosystems where interspecies interactions profoundly influence disease progression and patient prognosis [6, 7]. In particular, the co-existence of bacterial and fungal pathogens, such as *Staphylococcus* and *Candida* species, adds a layer of complexity to treatment regimens [8, 9]. Mixed *Candida*/bacterial infections account for >20% of all candidaemia episodes [10, 11]. The mortality rate is significantly higher in patients with mixed *Candida*/bacterial BSIs than in those with mono-candidaemia [12].

Interactions between *Staphylococcus aureus* and *Candida albicans* can be described as broadly synergistic. *S. aureus* can adhere to *C. albicans* hyphae and result in a dual-species biofilm [13]. Mixed *S. aureus/C. albicans* infections result in enhanced virulence and mortality in both murine and *Galleria mellonella* models [14–18]. Increased tolerance of *S. aureus* to antibiotics and *C. albicans* to antifungals such as miconazole have been reported [19–21]. However, not all interactions between *Candida* spp. and *S. aureus* are synergistic. Studies have shown that *S. aureus* supernatants can induce apoptosis in *Nakaseomyces (Candida) glabrata* [22].

While *C. albicans* is the most frequently isolated fungal species in the hospital setting worldwide, non-albicans *Candida* species including *N. glabrata*, *Candida tropicalis*, *Candida auris* and *Candida parapsilosis* are emerging [23–25]. Among these, *C. parapsilosis* has emerged as one of the three most frequent *Candida* species isolated from BSIs [26]. It is a leading cause of neonatal candidiasis, associated with significant morbidity in premature infants and is notably capable of horizontal transmission via the hands of healthcare workers, leading to documented outbreaks in neonatal intensive care units [27, 28].

Like other *Candida* species, *C. parapsilosis* can form a biofilm. However, this ability is strain-dependent, with the capacity to form biofilm also varying depending on the media used [29–33]. Despite its clinical importance, the interspecies interactions between *C. parapsilosis* and bacterial pathogens like *S. aureus* remain poorly defined. Several studies have investigated the effects of potential antimicrobials against *S. aureus* and *C. parapsilosis* mixed biofilms [34–36]. Other studies have focused on the specific conditions needed to produce mixed biofilms of *C. parapsilosis* with *S. aureus* [37] and *Staphylococcus epidermidis* [38]. However, very few studies have focused on the interactions that occur between *S. aureus* and *C. parapsilosis* in a mixed biofilm. It has been shown that *S. aureus* binds directly to *C. albicans* hyphae [13] during mixed biofilm formation. As *C. parapsilosis* does not make true hyphae, it is unknown how they may interact [29].

In this study, we investigated the interspecies dynamics between *C. parapsilosis* and *S. aureus* within a biofilm context. We report the discovery of small, heat-stable fungal-secreted factor(s) capable of both inhibiting and dispersing methicillin-sensitive *S. aureus* (MSSA) biofilms, although methicillin-resistant *S. aureus* (MRSA) remained recalcitrant. We demonstrate that this antagonistic effect is mediated by the modulation of environmental pH and a distinct transcriptomic reprogramming of the bacterial cell. These findings underscore the highly specific nature of fungal–bacterial interactions and highlight the potential for exploiting fungal secretomes to identify novel anti-biofilm strategies.

## Methods

### Strains and culture conditions

The bacterial and fungal strains used in this study are detailed in Table 1. Two MSSA isolates, DSM 799 and DSM 1104, were purchased from the DSMZ-German Collection of Micro-organisms and Cell Cultures GmbH. The MRSA isolate BH1CC [39] was kindly provided by Professor Eoghan O’Neill (Royal College of Surgeons in Ireland). All *S. aureus* and *C. parapsilosis* isolates were maintained on Tryptic Soy Agar (Oxoid) and Yeast Peptone Dextrose agar (Formedium), respectively, and stored at −80 °C in 15% (v/v) glycerol.

**Table 1.** List of strains used in this study

Isolate name	Abbreviation	Origin	Isolated from	Reference
*S. aureus* DSM 799	SA1	n/a	n/a	DSMZ
*S. aureus* DSM 1104	SA2	n/a	n/a	DSMZ
*S. aureus* BH1CC	SA3	–	–	[47]
CLIB214	CP1	Puerto Rico	Faeces	Type strain
CDC317	CP2	USA	Health care worker’s hand	Type strain
CDC173	CP3	USA	Blood or catheter culture	[75]
711701	CP4	Aberdeen, UK	Unknown	[76]
CDC167	CP5	USA	Blood or catheter culture	[75]
J961250	CP6	Lisbon, Portugal	Nail	[76]
CDC179	CP7	USA	Blood or catheter culture	[75]
J930733	CP8	Beerse, Belgium	Cat hair	[76]
103	CP9	London, UK	Anus	[76]
J930631/1	CP10	Africa	Cat hair	[76]
J960578	CP11	Hong Kong	Nail	[76]
81/040(S)	CP12	London, UK	Toe	[76]
81/041	CP13	Mayo Clinic, USA	Vagina	[76]
CDC177	CP14	USA	Blood or catheter culture	[75]
90–137	CP15	San Jose, USA	Orbital tissue	[76]
02–203	CP16	Bergamo, Italy	Blood	[76]
73/107	CP17	London, UK	Mouth	[76]
CDC165	CP18	USA	Blood or catheter culture	[75]
81/253	CP19	London, UK	Nail	[76]
81/040(C)	CP20	London, UK	Toe	[76]
J931058	CP21	Belgium	Nail	[76]
J951066	CP22	Korea	Nail	[76]
J950218	CP23	USA	Unknown	[76]
J931845	CP24	Japan	Unknown	[76]

### Standard biofilm quantification using a crystal violet assay

Biofilm biomass was quantified using a modified crystal violet (CV) staining method [40]. Following incubation in the appropriate media conditions, supernatants were aspirated to remove non-adherent cells, and wells were gently washed twice with 1× PBS. Adherent biomass was either air-dried or heat-fixed at 60 °C for 20 min. Biofilms were stained with 0.4% (wt/v) CV (Sigma-Aldrich) for 10 min at room temperature (RT), followed by two PBS washes to remove excess dye. Bound CV was solubilized in 33% (v/v) acetic acid for 15 min. Absorbance was measured at 595 nm using a Tecan Infinite M200 Pro plate reader. To maintain readings within the linear range, samples were diluted in PBS where necessary, and final values were adjusted by the appropriate dilution factor.

### Mixed-species co-culture biofilm assay

*S. aureus* and *C. parapsilosis* cells from overnight cultures in Tryptone Soya Broth (TSB) (Oxoid) were harvested by centrifugation, washed in PBS and adjusted to an OD_600_ of 0.1 and 1.0, respectively (~10^7^ c.f.u. ml^−1^) in TSB 0.2% glucose (TSB-0.2G). For mono-species controls, 100 µl of the adjusted cell suspension was combined with 100 µl of TSB-0.2G in Nunclon 96-well plates. For co-culture, 100 µl of each species suspension was added to the wells. Plates were incubated statically at 37 °C for 24 h prior to CV quantification.

### Preparation and characterization of *C. parapsilosis* cell-free supernatant

Fungal cell-free supernatant (CFS) was prepared by inoculating 10 ml of TSB-0.2G with the appropriate *C. parapsilosis* isolate and incubating overnight (~20 h) at 37 °C (180 r.p.m.). Cultures were centrifuged at 5,000 ***g*** for 10 min at 4 °C. The resulting supernatant was filter-sterilized (0.2 µm, Corning) and used immediately or stored at 4 °C. To assess thermal stability, CFS was boiled for 15 min prior to use. Size fractionation was performed using Pierce10 kDa molecular weight cut-off (MWCO) filters. Six millilitres of the fungal CFS was added to the filter and centrifuged at 5,000 ***g*** for 20 min at 4 °C. The filtrate (<10 kDa) and retentate (>10 kDa) were collected, with the retentate resuspended to the original volume in TSB-0.2G for subsequent assays.

### *C. parapsilosis* CFS biofilm assays

To assess the impact of *C. parapsilosis* CFS on *S. aureus* biofilm development, assays were performed in 24-well, 48-well or 96-well Nunclon plates. Wells contained either 100% TSB-0.2G (control) or a 1 : 1 mixture (v/v) of TSB-0.2G and fungal CFS. Cells from *S. aureus* overnight cultures were inoculated at a 1 : 100 dilution. Biofilm assays were incubated statically at 37 °C for 24 h prior to CV quantification.

#### Primary attachment assay

To assess the effect on primary attachment, assays were performed as described above but were terminated after 2 h of static incubation prior to CV quantification.

#### Biofilm dispersion

To assess the impact on mature biofilm, *S. aureus* biofilms were pre-established for 24 h in TSB-0.2G. Wells were washed twice with PBS before the addition of either 100% TSB-0.2G (control) or a 1 : 1 mixture (v/v) of TSB-0.2G and fungal CFS. Plates were incubated for a further 24 h at 37 °C prior to CV quantification.

### Dose-dependent biofilm inhibition

To determine the lowest concentration of *C. parapsilosis* CFS that has a statistically significant anti-biofilm effect on *S. aureus* biofilm development, assays were performed in 96-well Nunclon plates. Wells contained *S. aureus* cells diluted in TSB-0.2G (control) or decreasing concentrations of CFS/CP1 or CFS/CP6 (100–0.78%). Biofilm assays were incubated statically at 37 °C for 24 h prior to CV quantification.

### Glucose gradient biofilm assays

To evaluate the influence of glucose concentration on CFS activity, biofilm inhibition assays were performed under varying final glucose concentrations: baseline low glucose (~0.2% wt/v, TSB-0.2G), intermediate glucose (~0.5% wt/v, TSB-0.5G), and high glucose (~1.0% wt/v, TSB-1G). To maintain strict volumetric consistency across all experimental groups, wells were prepared via a 1 : 1 (v/v) mixing strategy as follows:

Untreated control environments for the intermediate and high-glucose conditions were established by combining sterile TSB supplemented with either 1.0% or 2.0% (wt/v) glucose 1 : 1 (v/v) with standard TSB-0.2G. CFS treatment groups were established by combining sterile TSB supplemented with either 1.0% or 2.0% (wt/v) glucose 1 : 1 (v/v) with raw fungal CFS (originally derived in TSB-0.2G). This matrix yielded a fixed final CFS concentration of 50% (v/v) while simultaneously establishing final glucose profiles of ~0.5 and 1.1% (wt/v), respectively.

Cells from *S. aureus* overnight cultures were inoculated at a 1 : 100 dilution. Biofilm assays were incubated statically at 37 °C for 24 h prior to CV quantification.

### *S. aureus* planktonic growth kinetics and cell viability assays

To determine the impact of *C. parapsilosis* CFS on *S. aureus* planktonic development, growth kinetics were monitored via OD_600_ tracking over a 24 h period. *S. aureus* overnight cultures were harvested, washed and adjusted to a baseline OD_600_ of 0.01 in either control medium (TSB-0.2G) or treatment medium (TSB-0.2G supplemented with 50% v/v *C*. *parapsilosis* CFS). Aliquots were transferred to a 96-well microtitre plate and incubated within a Varioskan LUX plate reader at 37 °C under continuous orbital shaking (180 r.p.m.). Planktonic growth was monitored automatically by recording OD_600_ values at hourly intervals.

Following the 24 h incubation period, cell viability was quantified via endpoint c.f.u. ml^-1^ determination. Cultures from each experimental condition were harvested, subjected to a 10-fold serial dilution series and drop-plated onto agar plates. Plates were incubated overnight at 37 °C, and visible colonies were enumerated to calculate absolute viable cell counts (c.f.u. ml^-1^).

### Biofilm supernatant pH measurements

Following 24 h of biofilm incubation, the culture supernatants were carefully aspirated, and the pH was measured using a calibrated pH probe. Measurements were performed for a minimum of three biological replicates across all experimental conditions.

## RNA extraction and transcriptomic sequencing

### Biofilm sample collection and RNA stabilization

Biofilms for transcriptomic analysis were established in six-well Nunclon plates using the conditions previously described in TSB-0.2G (mono-species *S. aureus,* dual-species *S. aureus* and *C. parapsilosis* cells and *S. aureus* treated with 50% CFS) or in TSB-1G (mono-species *S. aureus* and *S. aureus* treated with 50% CFS). Following 24 h of incubation, supernatants were removed, and wells were washed twice with PBS. Adherent biomass was harvested by mechanical scraping into 1 ml of PBS and immediately stabilized by addition to 2 ml of RNAprotect Bacteria Reagent (Qiagen). Samples were vortexed and incubated at RT for 5 min to ensure rapid arrest of transcription. Stabilized cells were harvested by centrifugation (5,000 ***g*** for 10 min at 4 °C), and pellets were either processed immediately or stored at −80 °C.

### Selective cell lysis and RNA purification

To ensure the isolation of bacterial RNA in dual-species samples, a selective lysis protocol was employed. Pellets were resuspended in TE buffer (30 mM Tris/Cl, 0.5 M EDTA, pH 8) supplemented with 50 µl of lysostaphin (1 mg ml^−1^; Sigma-Aldrich). Samples were incubated at 37 °C for 10 min with 10 s vortexing intervals every 2 min. For biofilms grown in TSB-1G, 50 µl of Proteinase K (20 mg ml^−1^; Qiagen) was added to facilitate the breakdown of the stabilized protein matrix. Total RNA was subsequently extracted using the RNeasy Mini Kit (Qiagen) following the manufacturer’s instructions. This selective lysis approach aimed to release bacterial RNA while maintaining the integrity of *C. parapsilosis* cells.

### RNA quality control

RNA concentration and purity were initially assessed using a NanoDrop 2000 spectrophotometer (Thermo Fisher Scientific); only samples with an A260/A280 ratio of above 2.0 and a concentration >50 ng /µl^−1^ were progressed. RNA integrity and the absence of fungal RNA contamination were confirmed using an Agilent 2100 Bioanalyzer (Agilent Technologies) or a Qubit 4 Fluorometer (Invitrogen). High-quality samples were stored at −80 °C.

### Library preparation and Illumina sequencing

Sample quality control, strand-specific library preparation and sequencing were performed by Novogene (Cambridge, UK). Sequencing was conducted on the Illumina platform using a 150 bp paired-end (PE150) strategy. Raw data were generated in FastQ format. The raw reads were processed through a bioinformatic pipeline to remove adapter sequences, low-quality reads (Phred score <Q30) and reads containing *N*>10% to generate clean reads for downstream differential expression analysis.

### Identification of differentially expressed genes and statistical analysis

RNA data analysis was carried out using the analysis platform Galaxy [41]. FASTQC (Galaxy Version 0.72+galaxy1) and MultiQC were used to assess the quality of the reads. Any reads containing adapter contamination were trimmed using Cutadapt. The reads were aligned to the reference genome of *S. aureus* NCTC 8325 (NCBI Reference Sequence: NC_007795) using HISAT2 (Galaxy Version 2.2.1+galaxy0) [42]. This reference genome was chosen as it has greater annotation than the DSM799 genome and increased compatibility with downstream analysis tools. Genes were initially annotated using SAOUHSC numbers and where possible this was converted to the gene name. Gene counts were generated by HtSeq-Count (Galaxy Version 0.9.1) using Union mode [43]. A counts matrix was produced and exported to R (version 4.4.0), where the EdgeR Bioconductor package was used for the annotation and creation of a differentially expressed gene (DEG) output table [44, 45]. Genes with an adjusted *P*-value<0.05 and an absolute Log2 fold change |(LogFC)|>1.5 were considered as being significantly differentially expressed. Figures depicting differential gene expression of *S. aureus* biofilms were created using the EdgeR and gplots R packages. All gene lists are provided in the supplementary material. The raw and processed RNA-sequencing data generated in this study have been deposited in the NCBI Gene Expression Omnibus database and are publicly accessible under the accession number GSE334781.

### General data and statistical analysis

All biofilm assays were carried out with a minimum of three biological replicates and four technical replicates for each condition. Graphs were created using GraphPad Prism 10. Significance values were calculated using a one-way ANOVA in GraphPad Prism 10.

## Results

### Interspecies co-culture with *C. parapsilosis* selectively reduces *S. aureus* biofilm biomass

To explore the interkingdom dynamics between *C. parapsilosis* and *S. aureus*, we initially evaluated the impact of co-cultivation on total biofilm biomass using the CV assay. A screen of 24 *C. parapsilosis* clinical isolates (CP1–24; Table 1) in TSB-0.2G revealed variable single-species biofilm-forming capabilities, with 12 *C. parapsilosis* isolates identified as biofilm-positive and 12 as biofilm-negative under these conditions (data not shown). From this cohort, two *C. parapsilosis* biofilm-deficient strains, CLIB214 (CP1) and CDC173 (CP3), and two *C. parapsilosis* robust biofilm-forming strains, J961250 (CP6) and CDC177 (CP14), were selected for further investigation in dual-species biofilm assays with the MSSA isolate *S. aureus* DSM799 (SA1).

**Fig. 1.**
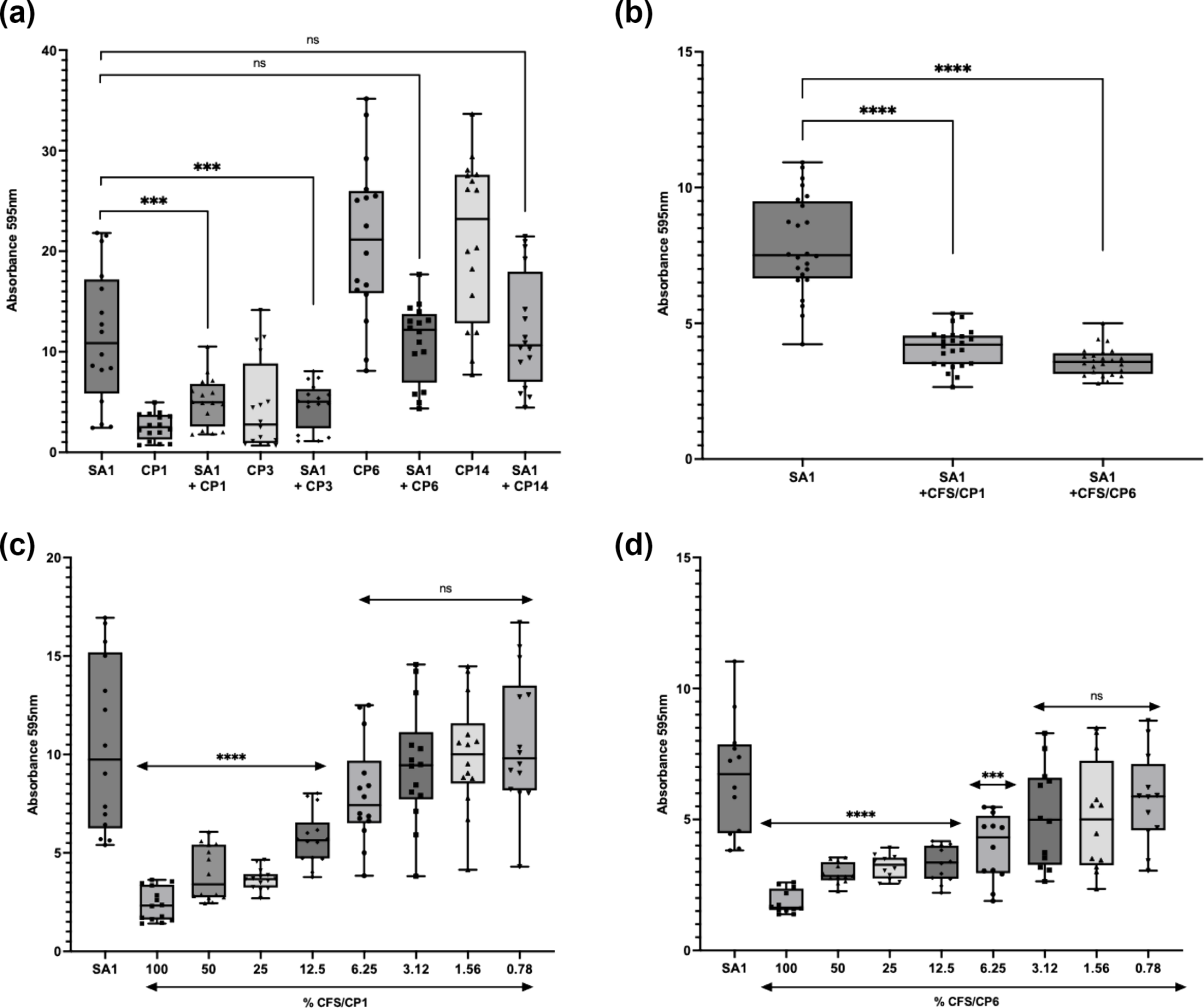
Effect of *C. parapsilosis* cells and CFS on *S. aureus* biofilm formation. Biofilm biomass (absorbance at 595 nm) was quantified via CV staining after 24 h incubation in TSB-0.2G. (**a**) Single- and mixed-species biofilms of *S. aureus* SA1 (DSM 799) with biofilm-deficient (*C. parapsilosis* CP1, CP3) or biofilm-positive (CP6, CP14) strains. (**b**) Maturation of *S. aureus* SA1 biofilms treated with 50% (v/v) CFS from strains CP1 or CP6. Dose-dependent inhibitory profiles of (**c**) CFS/CP1 and (**d**) CFS/CP6 across a decreasing concentration gradient (100–0.78%). Box plots display individual data points representing a minimum of three independent biological replicates. Statistical significance was determined by one-way ANOVA followed by multiple comparisons tests (***, *P*<0.001; ****, *P*<0.0001; ns, non-significant, *P*>0.05).

As expected, mono-species cultures of *C. parapsilosis* CP1 and CP3 failed to produce significant biofilm biomass in TSB-0.2G (Fig. 1a). Notably, co-culture of these strains with *S. aureus* SA1 resulted in a significant reduction in total biofilm biomass compared to the *S. aureus* mono-species control (*P*<0.001; Fig. 1a). In contrast, *C. parapsilosis* CP6 and CP14 produced robust mono-species biofilms (Fig. 1a). In co-culture with *S. aureus* SA1, the total biomass was not significantly different from the *S. aureus* mono-species control; however, a significant reduction in biomass was observed when compared to the fungal mono-species biofilms (Fig. 1a). These results indicate a complex, strain-specific interplay where the final mixed-species biofilm biomass is dictated by the inherent biofilm-forming capacity of the fungal partner. To determine if these antagonistic effects were mediated by secreted molecules, all subsequent experiments utilized *C. parapsilosis* CFSs.

### *C. parapsilosis* cell-free supernatant inhibits biofilm formation in a dose-dependent manner

To determine if the observed antagonism was mediated by secreted factors, we evaluated the effect of CFS from both biofilm-deficient (CP1) and biofilm-robust (CP6) *C. parapsilosis* strains named CFS/CP1 and CFS/CP6, respectively. In a 96-well plate assay, the addition of 50% (v/v) CFS/CP1 and CFS/CP6 significantly reduced *S. aureus* SA1 biofilm formation by 48 and 54%, respectively (*P*<0.0001; Fig. 1b).

The anti-biofilm activity of both secretomes exhibited a robust concentration-dependent profile, with a significant reduction in *S. aureus* biofilm formation initiated at concentrations >12.5% (v/v) for CFS/CP1 (*P*<0.0001) and >6.25% (v/v) for CFS/CP6 (*P*<0.001) relative to untreated controls (Fig. 1c, d)

**Fig. 2.**
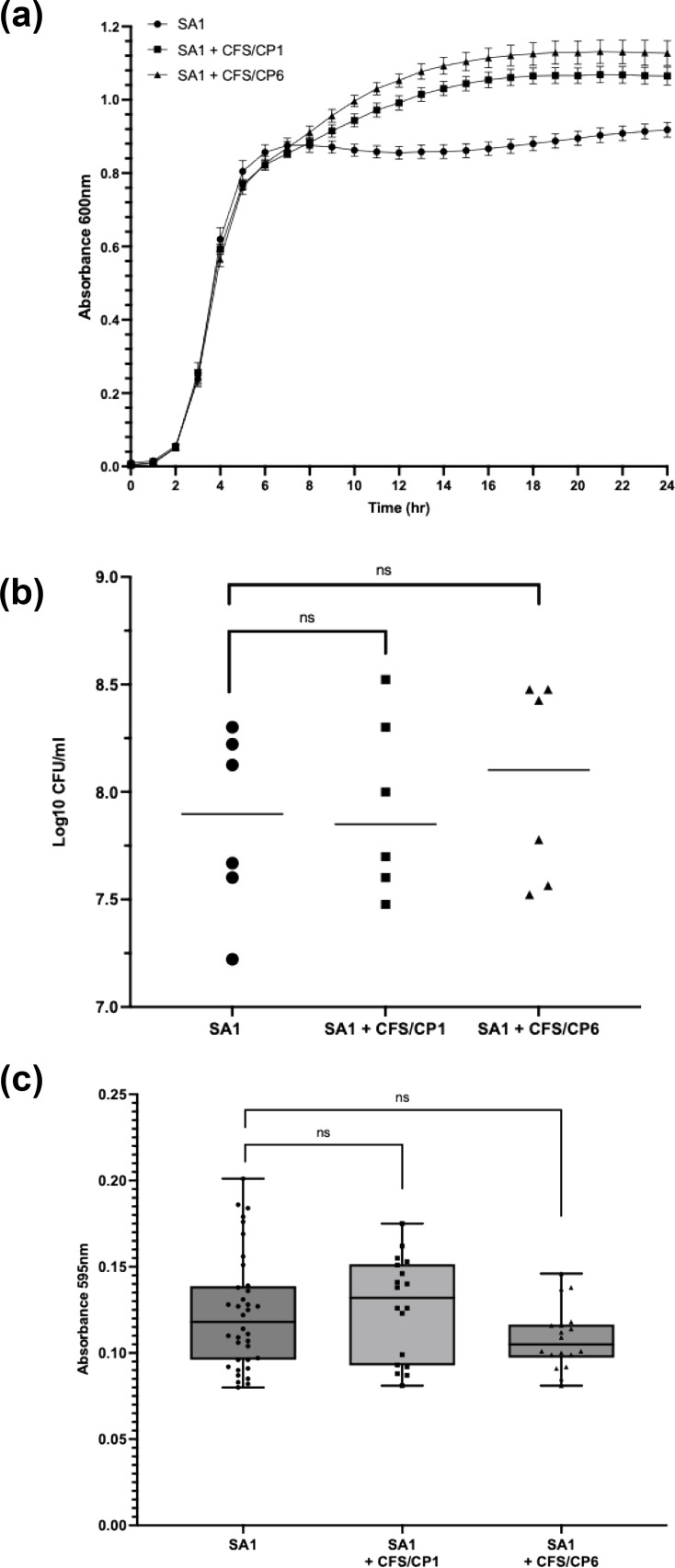
Effect of *C. parapsilosis* CFS on *S. aureus* planktonic growth, viability and primary attachment. (**a**) Planktonic growth kinetics (OD600) of *S. aureus* SA1 monitored over 24 h in TSB-0.2G supplemented with or without 50% (v/v) CFS/CP1 or CFS/CP6. (**b**) Absolute cell viability (log_10_ c.f.u. ml^-1^) quantified at the 24 h endpoint; horizontal lines represent the group means. (**c**) Primary surface attachment (absorbance at 595 nm) evaluated via CV staining following a 2 h incubation window. Individual data points represent a minimum of three independent biological replicates. Statistical analysis was performed using one-way ANOVA (ns, non-significant, *P*>0.05).

To ensure that the observed reduction in biofilm biomass was not a consequence of bactericidal activity or a suppression of cellular replication, *S. aureus* SA1 planktonic growth kinetics were assessed over a 24 h period (Fig. 2a). Generation time analysis during the exponential growth phase revealed no statistically significant differences between the conditions (*P*>0.05); the doubling time for *S. aureus* SA1 alone was 4.29±0.33 h, compared to 4.27±0.47 h in the presence of CFS/CP1 and 3.64±0.07 h with CFS/CP6.

Interestingly, area under the curve analysis revealed that the presence of either fungal secretome led to a significant increase in overall *S. aureus* SA1 turbidity (*P<*0.0001). This divergence was primarily observed during the late stationary phase of growth (Fig. 2a). Because the exponential generation times remained unaltered, this elevated stationary-phase absorbance suggests a metabolic shift or cellular aggregation phenotype rather than an accelerated replication rate.

To definitively confirm these kinetic observations, absolute cell viability was quantified at the 24 h endpoint via c.f.u. determination. In alignment with the exponential growth dynamics, viable planktonic cell counts remained statistically indistinguishable between the untreated control and both CFS-treated groups (*P*>0.05; Fig. 2b). Both treatment conditions maintained stable populations between ~7.9 and 8.1 log_10_ c.f.u. ml^-1^ (Fig. 2b). These data provide definitive validation that the secretome-mediated reduction in biofilm biomass is entirely independent of cellular death or bactericidal activity.

Furthermore, primary attachment assays revealed that the initial cellular adhesion of *S. aureus* SA1 after 2 h was entirely unaffected by the presence of 50% *C. parapsilosis* CFS (*P*>0.05; Fig. 2c). Collectively, these data establish that the secreted fungal factor specifically interferes with the matrix maturation phase of biofilm architecture rather than disrupting initial surface attachment, cellular replication or bacterial viability.

**Fig. 3.**
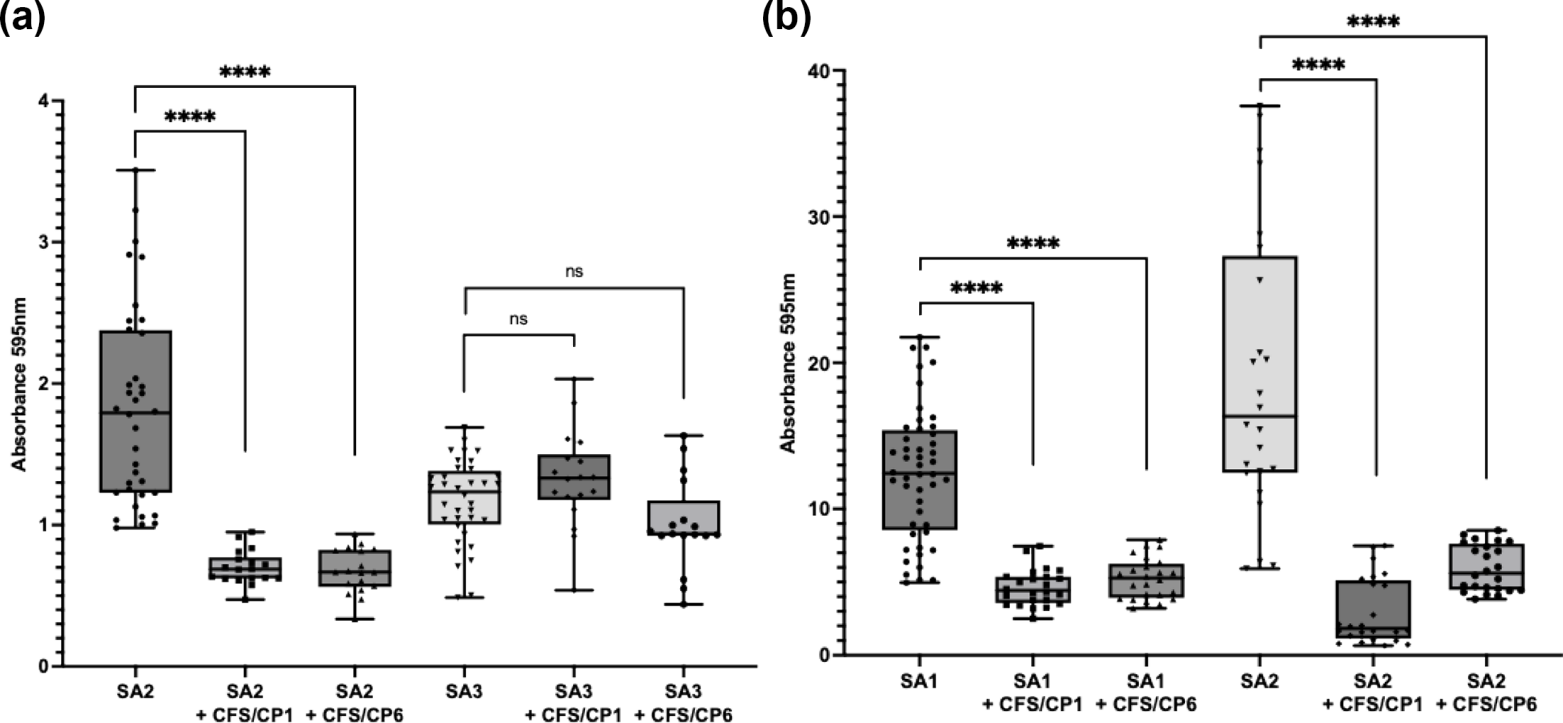
Effect of *C. parapsilosis* CFS on *S. aureus* biofilm formation and mature biofilm disruption. Biofilm biomass (absorbance at 595 nm) was evaluated via CV staining. (**a**) Biofilm maturation of MSSA isolate SA2 (DSM 1104) and MRSA isolate SA3 (BH1CC) following 24 h incubation with or without 50% (v/v) CFS from *C. parapsilosis* CP1 or CP6. (**b**) Active disruption of pre-established, 24 h mature biofilms of *S. aureus* SA1 and SA2 following treatment with fungal secretomes. Box plots display individual data points representing a minimum of three independent biological replicates. Statistical significance was determined by one-way ANOVA followed by multiple comparisons tests (****, *P*<0.0001; ns, non-significant, *P*>0.05).

The conservation of this anti-biofilm effect was tested against additional clinical isolates: MSSA *S. aureus* DSM1104 (SA2) and MRSA *S. aureus* BH1CC (SA3) [46, 47]. Fungal CFS significantly inhibited *S. aureus* SA2 biofilm formation, yielding biomass reductions of 62% for CFS/CP1 and 63% for CFS/CP6 (*P*<0.0001; Fig. 3a). In alignment with our findings for SA1, kinetic tracking and endpoint viability assays confirmed that this disruption occurred independently of any planktonic growth defects or bactericidal activity against SA2 (Fig. S1, available in the online Supplementary Material). Notably, however, neither CFS/CP1 nor CFS/CP6 exerted any inhibitory effect on the MRSA isolate SA3 under identical experimental conditions (*P*>0.05; Fig. 3a). This highlights a distinct, strain-specific susceptibility to the fungal secretome and suggests that underlying variations in matrix composition or genetic background govern resistance to the secretome’s activity.

Finally, we examined the capacity of the *C. parapsilosis* CFS to disrupt pre-established *S. aureus* biofilms. Treatment of mature (24 h) biofilms with 50% *C. parapsilosis* CFS resulted in substantial dispersal of both *S. aureus* SA1 and SA2 biomass. Specifically, CFS/CP1 reduced established SA1 and SA2 biofilms by 63 and 85%, respectively, while CFS/CP6 resulted in 58 and 69% reductions (*P*<0.0001; Fig. 3b). These results demonstrate that the *C. parapsilosis* secretome contains potent bioactive molecules capable of both preventing biofilm formation and destabilizing mature *S. aureus* communities.

### The anti-biofilm activity of *C. parapsilosis* CFS is abrogated across a glucose gradient due to loss of environmental pH modulation

**Fig. 4.**
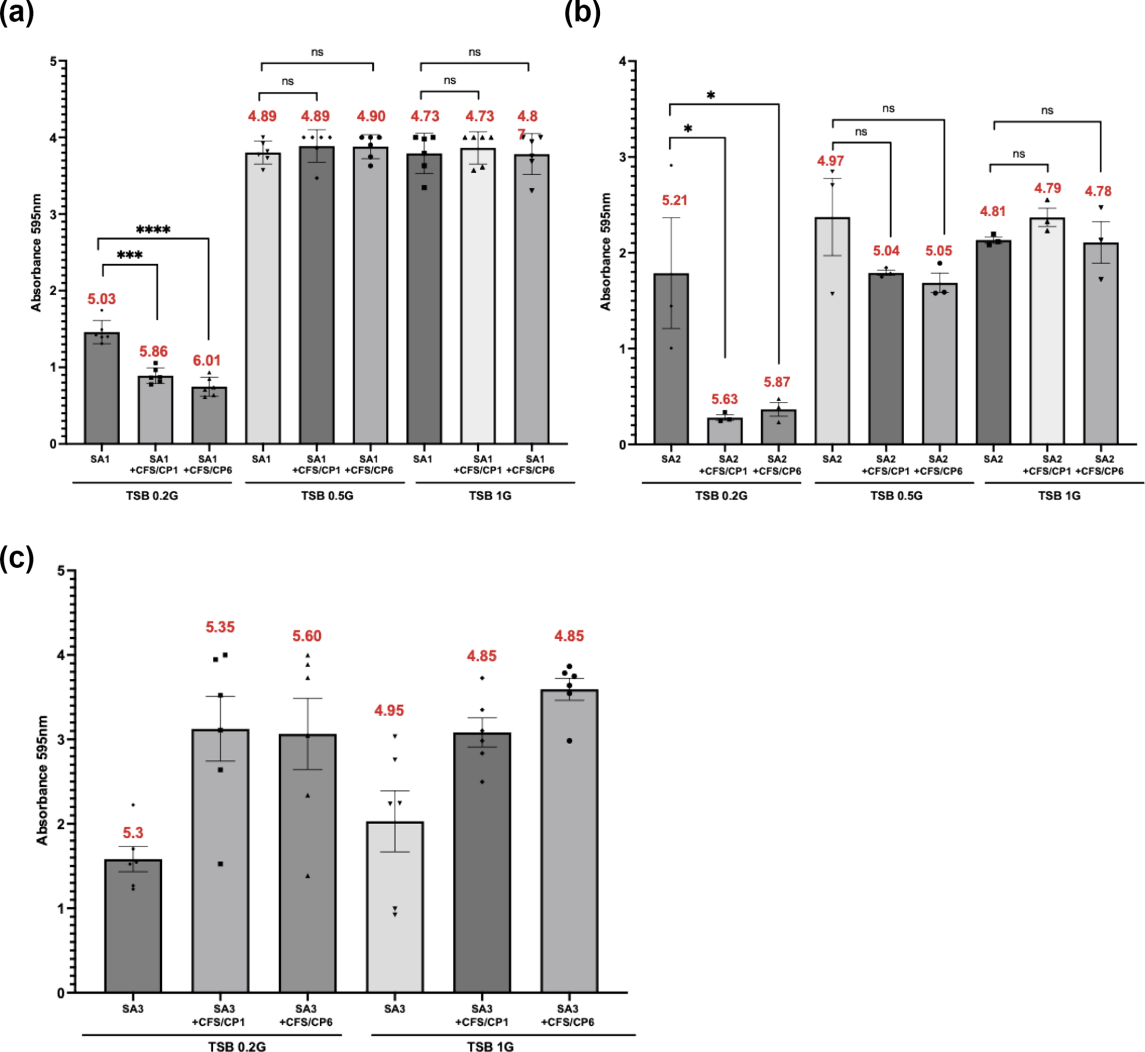
Influence of glucose concentrations on *C. parapsilosis* CFS anti-biofilm activity and microenvironmental pH. Biofilm biomass (absorbance at 595 nm) was quantified after 24 h incubation in the presence or absence of 50% (v/v) CFS from *C. parapsilosis* strains CP1 or CP6 under varying glucose availability. Profiles are shown for *S. aureus* clinical isolates (**a**) SA1, (**b**) SA2 and (**c**) SA3. Final extracellular microenvironmental pH values measured at the 24 h endpoint are indicated in red text above each corresponding treatment bar. Data represent the mean±sem of independent biological replicates. Statistical significance was determined using one-way ANOVA followed by Dunnett’s multiple comparisons test (*, *P*<0.01; ***, *P*<0.001; ****, *P*<0.0001; ns, non-significant *P*>0.05).

To evaluate the influence of carbohydrate availability and carbon catabolite repression on fungal secretome anti-biofilm activity, biofilm maturation assays were performed across a tight glucose gradient: baseline low glucose (TSB-0.2G), intermediate glucose (TSB-0.5G) and high glucose (TSB-1G). The anti-biofilm potency of *C. parapsilosis* CFS proved strictly dependent on the glucose concentration of the growth medium. Consistent with our initial findings, 50% (v/v) CFS from both CP1 and CP6 reduced *S. aureus* SA1 and SA2 biofilm (*P*<0.01–0.0001) under standard low-glucose conditions (TSB-0.2G) (Fig. 4a, b).

Strikingly, this inhibitory phenotype was completely abolished as carbohydrate levels scaled up. In both the intermediate TSB-0.5G and high TSB-1G environments, both *S. aureus* SA1 and SA2 formed robust, dense biofilms regardless of the presence of fungal CFS, displaying no statistically significant reductions in biomass compared to their respective untreated controls (*P*>0.05; Fig. 4a, b).

To elucidate the physiological mechanism driving this nutrient-dependent block, environmental pH values were recorded from the biofilm supernatant immediately following the 24 h incubation period (denoted in red; Fig. 4). Prior to inoculation, the baseline pH across all media configurations (TSB-0.2G, 0.5G and 1G) and the cell-free secretomes was confirmed to be uniformly near-neutral, at a collective average of 6.90±0.16. Following the 24 h biofilm assay, distinct shifts from this baseline were observed.

In the low-glucose environment (TSB-0.2G), a distinct and specific pH-buffering effect was observed in wells treated with fungal secretomes. While untreated *S. aureus* SA1 and SA2 control biofilms strongly acidified the media down to pH 5.03 and 5.21, respectively, the addition of CFS from either CP1 or CP6 maintained the environment at a significantly less acidic, near-neutral window ranging from 5.86 to 6.01 for SA1 (Fig. 4a) and 5.63 to 5.87 for SA2 (Fig. 4b). One-way ANOVA confirmed that these secretome-mediated alterations in environmental pH were highly statistically significant (*P*<0.001–0.0001) for both susceptible isolates relative to their untreated controls.

Conversely, escalating the background glucose availability completely overwhelmed this protective buffering signature. In both TSB-0.5G and TSB-1G, the pH decreased and remained fixed within a narrow range of 4.89–4.90 for SA1 and 4.97–5.05 for SA2 in the intermediate media, and plummeted further to 4.73–4.80 (SA1) and 4.78–4.81 (SA2) under high-glucose conditions, completely erasing any statistical or physical differences between untreated controls and CFS-treated groups (Fig. 4a, b)

To further investigate the role of environmental pH modulation, the resistant MRSA isolate *S. aureus* SA3 was subjected to identical baseline and high-glucose conditions (Fig. 4c). In direct contrast to the susceptible isolates, the addition of fungal CFS failed to inhibit SA3 biofilm formation even in the low-glucose environment (TSB-0.2G), with biomass values significantly increasing upon treatment. Interestingly, this resistance directly correlated with a failure to mirror the protective buffering signature seen in SA1 and SA2. Under low-glucose conditions (TSB-0.2G), the extracellular milieu of SA3 remained highly acidic, sitting at pH 5.30 for the untreated control and shifting only minimally to 5.35 following CFS/CP1 treatment. A slight buffering effect was observed with CFS/CP6, though the environment remained distinctly acidic at pH 5.60. Under high-glucose conditions (TSB-1G), the environment acidified completely down to a baseline floor (4.85 to 4.95) across all groups (Fig. 4c).

Collectively, these data suggest that while the anti-biofilm activity of the *C. parapsilosis* secretome is intrinsically linked to its ability to modulate or maintain environmental pH, this protective mechanism is bypassed under high-glucose conditions and actively overridden by unknown, strain-specific factors.

### The *C. parapsilosis* anti-biofilm activity is mediated by small, heat-stable molecules

To characterize the biochemical properties of the active anti-biofilm component(s), we performed a series of stability and fractionation assays of CFS/CP1 and CFS/CP6. The anti-biofilm effects of these were tested against *S. aureus* SA2, which was selected for its capacity to form robust and highly reproducible biofilms.

**Fig. 5.**
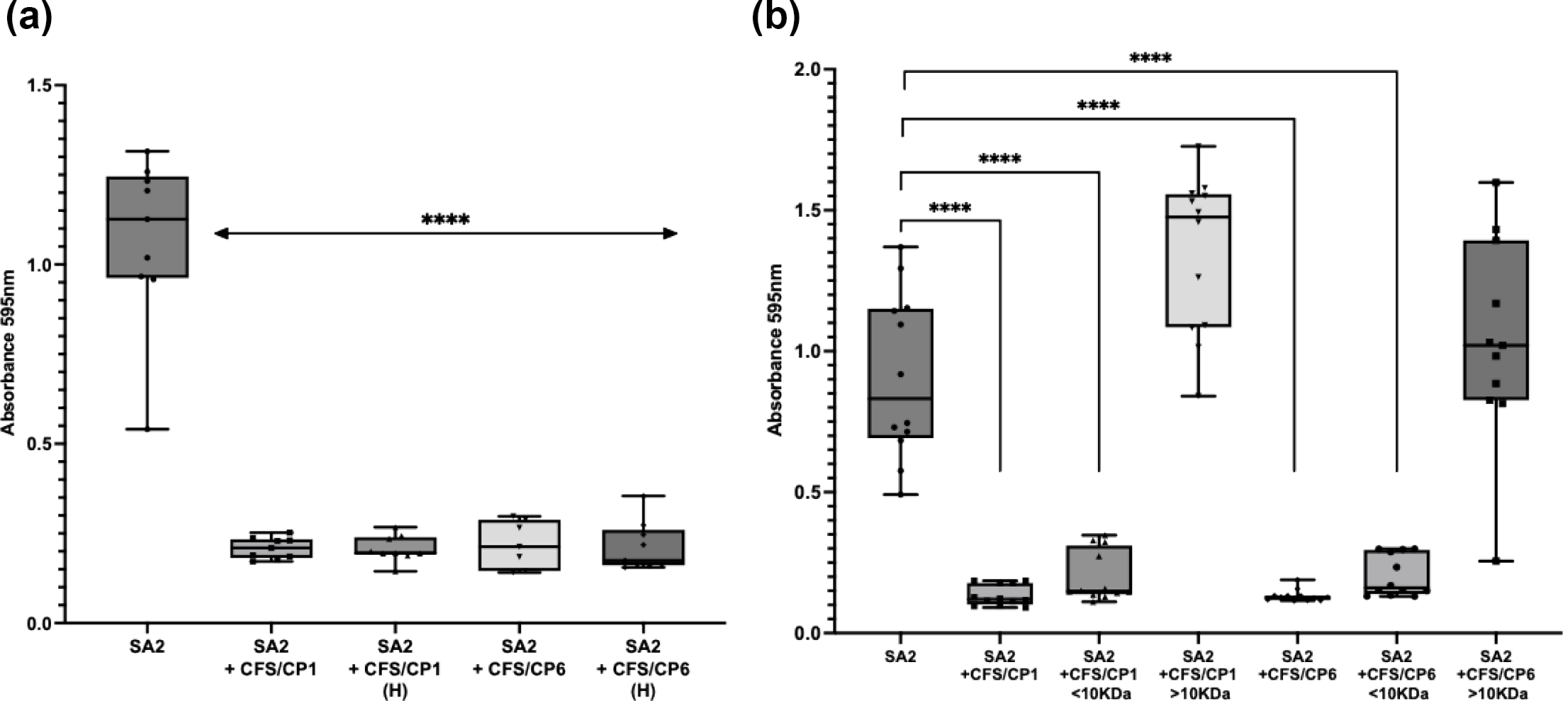
Characterization of the biochemical properties of *C. parapsilosis* CFS. Biofilm biomass (absorbance at 595 nm) of *S. aureus* SA2 was quantified via CV staining after 24 h. (**a**) Thermal stability profiling of *C. parapsilosis* secretomes; assays were supplemented with untreated CFS or CFS subjected to boiling for 15 min [denoted as (**h**)]. (**b**) Size-fractionation profiling using 10 kDa MWCO filters; assays were treated with unfractionated CFS, the <10 kDa filtrate, or the >10 kDa retentate from strains CP1 or CP6. Box plots present individual data points from a minimum of three independent biological replicates. Statistical significance was evaluated by one-way ANOVA followed by multiple comparisons tests (****, *P*<0.0001).

First, we assessed the thermostability of the fungal secretome by boiling the CFS from both *C. parapsilosis* CP1 and CP6 for 15 min. As demonstrated in Fig. 5a, heat treatment did not alleviate the inhibitory activity of the supernatants. Biofilm formation by *S. aureus* SA2 remained significantly reduced by ~80% (*P*<0.0001) regardless of whether the CFS was heat treated or remained at RT. The retention of activity following thermal stress suggests that the bioactive factor(s) are likely not proteinaceous in nature.

To determine the approximate size of the inhibitory molecule(s), CFS samples were subjected to size-exclusion fractionation using 10 kDa MWCO filters. The resulting filtrate (<10 kDa) and retentate (>10 kDa) were tested for anti-biofilm activity against *S. aureus* SA2. The results indicated that the inhibitory phenotype was localized exclusively to the <10 kDa fraction (Fig. 5b). This fraction, along with the unfractionated crude CFS, effectively inhibited biofilm maturation, whereas the >10 kDa retentate had no significant impact on biomass development (Fig. 5b). Taken together, these data characterize the *C. parapsilosis* anti-biofilm factor(s) as small, heat-stable molecule(s), providing a foundation for future structural and chemical identification.

### Transcriptomic profiling reveals a fungal-induced metabolic trap in *S. aureus*

To elucidate the molecular mechanisms underlying *S. aureus* biofilm inhibition, we performed comparative transcriptomic analysis (RNA-seq) of *S. aureus* SA1 biofilms co-cultured with *C. parapsilosis* CLIB214 CP1 cells or treated with 50% (v/v) CFS from this fungal strain (CFS/CP1). To ensure the purity of the bacterial transcriptome in mixed-biofilm co-cultures, a selective lysis strategy was employed; biofilms were treated with lysostaphin to specifically release *S. aureus* RNA, leaving the yeast cells intact. The absence of fungal RNA contamination was confirmed by Bioanalyzer analysis (data not shown).

**Fig. 6.**
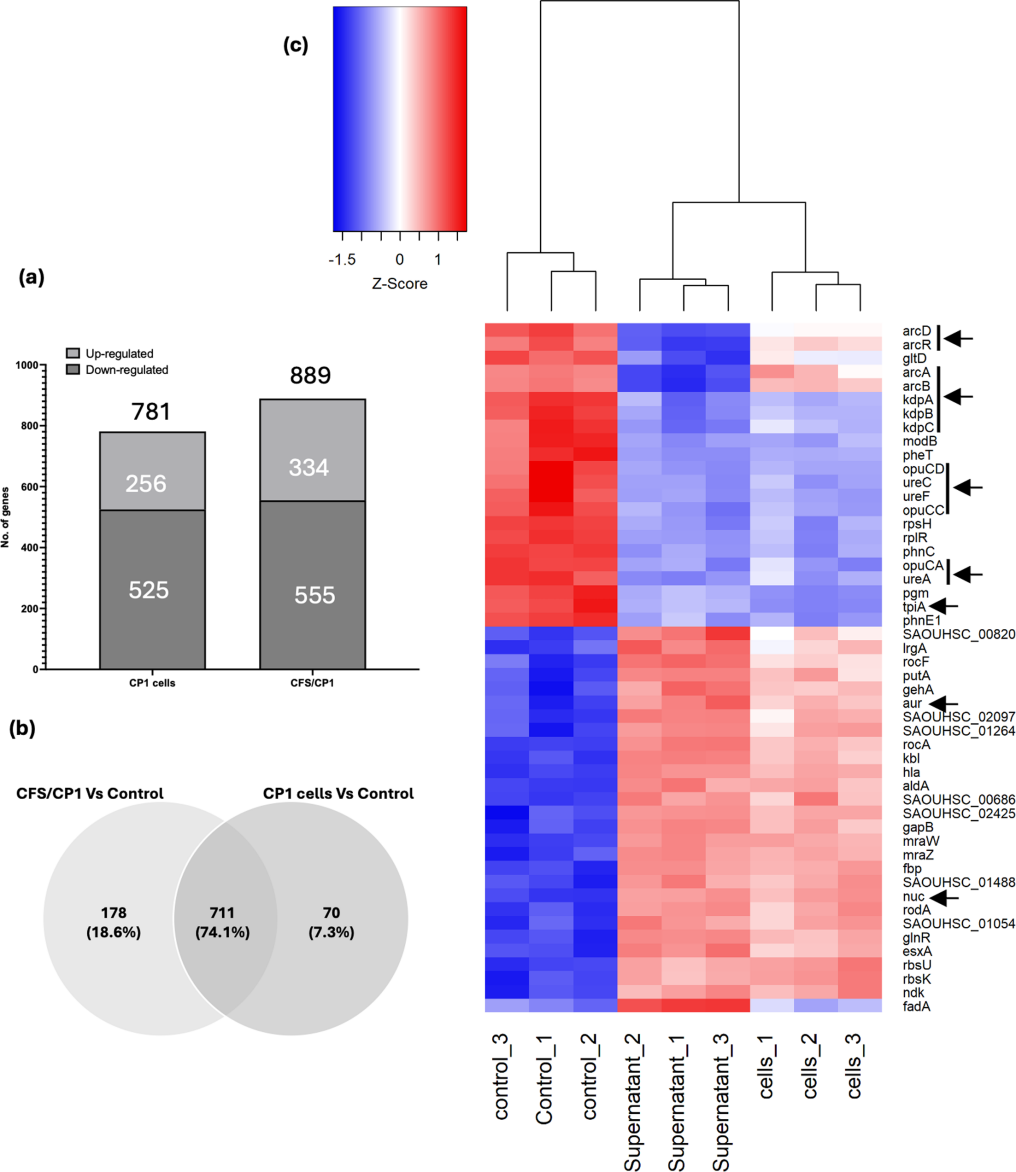
Transcriptional profiling of *S. aureus* SA1 (DSM 799) exposed to *C. parapsilosis* CP1 cells or CFS/CP1. RNA-sequencing was performed on *S. aureus* SA1 biofilms grown for 24 h in TSB-0.2G under single-species control conditions, co-cultured with *C. parapsilosis* CP1 cells, or treated with 50% (v/v) CFS/CP1. (**a**) Total number of significantly DEGs, segmented by up-regulation and down-regulation, relative to the untreated control. (**b**) Venn diagram showing the distribution and percentage overlap of shared and unique DEGs between the cell and CFS exposure conditions. (**c**) Hierarchical clustering and heatmap analysis displaying relative transcriptional expression levels (Z-scores) of selected key staphylococcal target genes across independent biological replicates (*n*=3). Colour gradient indicates relative up-regulation (red) or down-regulation (blue).

In TSB-0.2G, significant transcriptomic reprogramming was observed in both CP1 co-cultures and CFS/CP1-treated biofilms (|log2FC|>1.5, *P*<0.05). In co-culture assays, 781 DEGs were identified with 256 upregulated and 525 downregulated. CFS/CP1-treated biofilm cells resulted in 889 DEGs with 334 upregulated and 555 downregulated (Fig. 6a, supplementary Tables S1 and S2). We identified a substantial overlap of 711 differentially DEGs common to both conditions (Fig. 6b, supplementary S3). The expression levels of these core genes were highly correlated (r=0.97, *P*<2.2e-16) confirming that the fungal cell-free secretome largely recapitulates the effects of live fungal co-culture.

To gain an initial overview of the transcriptomic shift, a heatmap of the top 50 DEGs was generated. This analysis revealed that the transcriptional responses of *S. aureus* to both *C. parapsilosis* CLB214 (CP1) cells and the CFS from this strain (CFS/CP1) were highly similar to each other and remained distinct from the control (Fig. 6c). Notably, genes essential for acid and stress tolerance were significantly downregulated in both fungal-exposed conditions (Fig. 6c). The heatmap also highlighted a robust induction of genes involved in biofilm matrix degradation, specifically the thermonuclease gene (*nuc*) and the extracellular metalloprotease aureolysin (*aur*). Conversely, metabolic genes such as triosephosphate isomerase (*tpiA*) were downregulated compared to the control (Fig. 5c).

While the heatmap provided a snapshot of the most significant changes, a broader analysis of the 711 overlapping DEGs revealed a more complex, pleiotropic effect on the *S. aureus* SA1 transcriptome (Table 2, supplementary S3). Consistent with the observed biofilm-deficient phenotype, the *ica* operon – responsible for the production of polysaccharide intercellular adhesin (PIA) – was significantly downregulated, as were genes encoding critical surface adhesins such as fibronectin-binding protein A (*fnbA*) (Table 2). Parallel to the suppression of matrix synthesis, genes involved in active matrix degradation were highly upregulated, including *nuc* and the *spl* (serine protease-like) operon (Table 2). Furthermore, a significant downregulation of genes involved in glucose metabolism and the acid stress response was observed (Table 2).

**Table 2.** Key functional categories of DEGs found in the overlap between CFS/CP1 versus control and CP1 versus control

Biological category	Representative gene	Expression trend
Biofilm matrix formation	*icaA, icaB, icaC, sasD, fnbA*	Downregulated
Matrix degradation	*nuc, aur, gehA, splA/B/C/D/E/F, scpA.1*	Upregulated
Carbon metabolism	*gapA, pgk, tpiA, pgm, eno*	Downregulated
Acid and stress tolerance	arcA/B/C/D/R, ureA/B/C/D/F/G, opuCA/B/C/D, kdpA/B/C	Downregulated
Alternative carbon scavenging	*putA, rocA, aldA, fadA, kbl, hutG, rbsK*	Upregulated
Gluconeogenesis	*gapB, fbp, pckA*	Upregulated
Toxins and virulence	*hla, hlgA, lukD/E/H, esxA*	Upregulated

Consistent with our phenotypic observations, the extensive transcriptomic reprogramming induced by *C. parapsilosis* CFS was entirely abolished under high-glucose conditions (TSB-1G). Comparative transcriptomic analysis of *S. aureus* treated with CFS/CP1 in the presence of 1% glucose revealed a complete absence of DEGs (0 DEGs) relative to the untreated TSB-1G control. This absolute lack of transcriptional alteration confirms that elevated glucose concentrations completely render *S. aureus* recalcitrant to fungal-mediated genetic manipulation. By locking the bacteria in its baseline glycolytic metabolic state, the presence of excess glucose completely neutralizes the regulatory cues of the secretome, preventing the activation of matrix-degrading pathways and preserving the integrity of the biofilm

## Discussion

Cross-kingdom interactions within polymicrobial biofilms are governed by a complex language of secreted metabolites and dynamic environmental cues [7, 48]. While the synergistic, cooperative relationship between *C. albicans* and *S. aureus* within mixed infectious niches is well-documented [8], in this study we observed a distinct antagonism mediated by the *C. parapsilosis* secretome.

Our findings demonstrate that *C. parapsilosis* produces heat-stable, low-molecular-weight factor(s) (< 10 kDa) capable of both robustly inhibiting *S. aureus* biofilm maturation and actively dispersing established biomass (Figs. 1 and 3). This inhibition was not due to any growth defect or alterations to bacterial primary attachment suggesting an effect on biofilm maturation and matrix production (Fig. 2). These data confirm that the fungal secretome exerts a non-bactericidal, non-biocidal mechanism that selectively interferes with the architecture of the matrix during the biofilm maturation phase rather than suppressing bacterial viability or initial surface attachment.

Our phenotypic profiling across a tight glucose gradient (Fig. 4) reveals that the secretome relies on a conditional ‘Metabolic Trap’ model. In carbon-limited media (TSB-0.2G), the fungal CFS exerts a potent buffering effect on susceptible MSSA strains (SA1 and SA2), preventing microenvironmental acidification and maintaining a near-neutral pH (5.63–6.01) compared to the highly acidic untreated controls (pH 5.03–5.21). However, escalating glucose to intermediate (TSB 0–5G) or high (TSB-1G) levels drives accelerated staphylococcal glycolytic flux. The resulting surge in acidic metabolites overpowers the secretome’s buffering capacity, plummets the pH below 5.05 and completely abolishes the anti-biofilm phenotype (Fig. 4a, b). This metabolic checkpoint partially explains the resistance of MRSA isolate SA3; even in low glucose, SA3 fails to undergo environmental buffering and maintains an acidic milieu (pH 4.95–5.35) that preserves its biofilm matrix (Fig. 4c).

Todd *et al.* previously reported that planktonic co-cultures of *S. aureus* with *C. albicans, C. tropicalis* or *Candida krusei* drive environmental alkalinization, whereas *C. parapsilosis* induces only a modest pH shift [49]. Crucially, that alkalinization was attributed directly to active fungal cellular metabolism rather than a stable secreted molecule [49]. Our study extends these observations to a biofilm model, proving that *C. parapsilosis* actively synthesizes and secretes cell-free factors capable of counteracting *S. aureus*-mediated acidification. However, we demonstrate that this protective interaction is strictly conditional, regulated by both background glucose concentrations and the specific genetic background of the staphylococcal strain.

Our transcriptomic data provides a clear molecular blueprint for this ‘Metabolic Trap’. Exposure to the fungal secretome triggers a profound down-regulation of primary staphylococcal glycolytic machinery, significantly suppressing core genes including *tpiA* (triosephosphate isomerase), *gapA* (glyceraldehyde-3-phosphate dehydrogenase), *pgk* (phosphoglycerate kinase), *pgm* (phosphoglyceromutase) and *eno* (enolase) (Fig. 6c). As these enzymes are critical for maintaining glycolytic flux, their inhibition likely reduces the rate of glucose metabolism, preventing the rapid environmental acidification typically seen in maturing *S. aureus* biofilms. This is a critical shift, as high glycolytic activity has been directly linked to increased biofilm biomass [50].

This reduced metabolic rate maintains the local environment within a near-neutral range (pH 5.86–6). Consequently, *S. aureus* fails to induce the acid-defence systems essential for pH homeostasis, as evidenced by the profound downregulation of the arginine deiminase (ADI), urease and Kdp systems. The ADI pathway (comprising *arcA, arcB, arcC, arcD* and the regulator *arcR*) and the urease operon (*ureABC*) are primary survival mechanisms that produce ammonia to neutralize the cytoplasm and local environment [51]. Previous studies confirm that *arc* genes are typically upregulated during biofilm formation [52, 53], and their inactivation results in marked biofilm decreases [54]. Furthermore, the downregulation of the arginine-ornithine antiporter *arcD* is particularly significant, as its deletion has been shown to decrease the deposition of PIA [55].

The significant downregulation of the urease operon (*ureA-G*) further supports a model of disrupted pH homeostasis. This repression may be mediated by the global regulator *mgrA*, which was upregulated in our study. MgrA is known to prevent *S. aureus* from mounting a standard acid-defence response [56] and has been shown to repress the *ica* operon, which is required for MSSA biofilm formation [57]. Similarly, the downregulation of the *kdpABC* operon suggests that the treated bacteria are not experiencing the ionic demands or transmembrane electrochemical gradients usually associated with acidic biofilm maturation [58].

Crucially, the fungal CFS appears to trigger matrix degradation. Among the top DEGs was *nuc*, which encodes a thermonuclease that degrades eDNA to structure the biofilm during the exodus stage [59, 60]. We also observed a significant downregulation of the *ica* operon [53]. Because nuclease activity is optimized at a buffered pH of 5.86–6 [61], the fungal-induced pH shift creates the ideal conditions for eDNA degradation. We propose that by simultaneously upregulating *nuc* and downregulating *ica*-mediated PIA production, the fungal CFS prevents the essential interaction between PIA and eDNA required for aggregation [62], thereby forcing a premature exodus that destabilizes the biofilm.

Collectively, these data support our proposed ‘Metabolic Trap’ model to explain the antagonism of *C. parapsilosis* against *S. aureus* under low-glucose conditions. In this model, the fungal secretome triggers a glycolytic shift that prevents environmental acidification. This tricks *S. aureus* into downregulating its acid-tolerance systems while maintaining a near-neutral pH that facilitates high nuclease activity. By manipulating the metabolic environment, the fungal CFS forces the bacteria into an exodus phase, leading to the degradation of the biofilm matrix and a failure to establish a stable biofilm architecture.

It should be noted that a reference genome of *S. aureus* NCTC 8325 (NCBI Reference Sequence: NC_007795) was chosen for this transcriptomic analysis as it provides a higher degree of functional annotation than the native DSM799 draft genome, ensuring increased compatibility with downstream bioinformatic analysis tools. However, using a reference genome could naturally introduce localized alignment biases or overlook highly strain-specific accessory genes that may exist within the DSM799 isolate.

The transcriptomic profile of *S. aureus* under high-glucose conditions (TSB-1G) further validates the ‘Metabolic Trap’ hypothesis by demonstrating a failure of the fungal CFS to induce the characteristic metabolic shift. This is observed by the total absence of DEGs (0 DEGs) observed when treating *S. aureus* with CFS under high-glucose conditions (TSB-1G). In high glucose, the bacteria maintain baseline glycolytic flux, upregulate acid-defence systems and remain completely recalcitrant to secretome reprogramming, perfectly validating our phenotypic model.

The anti-biofilm effect of *C. parapsilosis* CFS was not universal: while MSSA isolates DSM 799 (SA1) and DSM 1104 (SA2) were highly susceptible, the MRSA isolate BH1CC (SA3) remained entirely unaffected (Fig. 3a). This divergence is likely rooted in the fundamental biochemical disparities governing MSSA and MRSA extracellular biofilm matrix architectures [63]. Traditional MSSA strains predominantly rely on an *ica*-dependent, PIA matrix framework cross-linked with extracellular DNA (eDNA). Conversely, many clinical MRSA isolates, particularly healthcare-associated (HA-MRSA) lineages such as the recalcitrant BH1CC (SA3) strain utilized here, frequently repress or bypass the *ica* operon, assembling protein-dominated matrices heavily enriched with fibronectin-binding proteins (FnBPs), Protein A and biofilm-associated protein (Bap) [47, 64–66]. Our finding suggests that while the fungal secretome can trigger a dispersal-like response in *S. aureus*, the structural target of this response is absent in protein-mediated biofilms, effectively rendering MRSA isolates immune to the anti-biofilm effect of the fungal secretome.

This structural divergence perfectly aligns with our proposed 'Metabolic Trap' model. Our transcriptomic data revealed that the fungal CFS acts on susceptible strains by downregulating *ica*-mediated PIA production and simultaneously maintaining a near-neutral pH environment that optimizes host thermonuclease (*nuc*) activity to degrade biofilm associated eDNA. Because the structural integrity of the BH1CC (SA3) biofilm relies on a dense proteinaceous scaffold rather than an electrostatic PIA-eDNA network, it lacks the primary biomolecular targets of this fungal-induced dispersal response [39]. Furthermore, our phenotypic profiling (Fig. 4c) demonstrated that SA3 fails to undergo environmental buffering even under low-glucose conditions, maintaining a highly acidic extracellular milieu (pH 4.95–5.35) that naturally suppresses nuclease activity. Consequently, the structural immunity of its protein-mediated matrix, combined with its distinct metabolic resistance to environmental buffering, renders this MRSA isolate entirely immune to the anti-biofilm mechanisms of the fungal secretome.

The *C. parapsilosis* CFS, like other microbial supernatants, is composed of a mixture of secreted proteins, metabolites and small molecules [67]. In this study, size-fractionation and heat-stability assays identified the active component(s) to be highly heat-resistant and under 10 kDa in size (Fig. 5a, b), strongly pointing to a non-proteinaceous nature. This is consistent with findings by Glatthardt *et al*. [68], who demonstrated that *S. epidermidi*s secretes small, heat-stable molecules (3–10 kDa) capable of disrupting *S. aureus* biofilms [68]. The *C. albicans* quorum-sensing (QS) molecule farnesol is known to interact with and inhibit *S. aureus* [69–71]. While historical interkingdom studies often centre on traditional fungal QS signals like farnesol or tyrosol, which are natively produced at much lower baseline levels by *C. parapsilosis* compared to *C. albicans* [72, 73] – our data suggest a distinct biochemical mechanism driven primarily by metabolic subversion.

Given our concurrent phenotypic and transcriptomic findings, it is highly probable that the secretome contains specific active metabolites that directly intercept central staphylococcal pathways. By altering glucose catabolism or aberrantly downregulating the arginine/ADI pathway, these fungal molecules trick *S. aureus* into an altered metabolic state that systematically prevents the environmental acidification required for normal matrix development. It remains to be determined whether this anti-biofilm phenotype is driven by a single, novel bioactive moiety or the synergistic effect of multiple concurrent fungal metabolites. Future work will focus on characterizing the exact chemical identity and precise biochemical mode of action of these purified compounds.

From a clinical and translational perspective, the discovery of a non-bactericidal, secretome-mediated mechanism of staphylococcal biofilm disruption carries profound relevance within the current landscape of the global AMR crisis. Polymicrobial, device-associated biofilms are notoriously recalcitrant to standard, high-dose bactericidal therapies due to the physical shielding afforded by the extracellular matrix, which frequently drives the evolutionary selection of multi-drug-resistant strains [74]. Emerging anti-biofilm strategies have increasingly shifted focus away from traditional bactericidal agents and toward non-lethal dispersal mechanisms, such as small-molecule signalling inhibitors or matrix-degrading enzymes, which destabilize the biofilm architecture without exerting strong evolutionary pressure for survival. Our data identifies a critical metabolic vulnerability in *S. aureus* that could be exploited through site-specific environmental manipulation to disrupt biofilm architecture and restore bacterial vulnerability to conventional antimicrobials. These findings demonstrate that while exploiting interkingdom interactions as therapies remains a challenge, they provide a promising blueprint for next-generation anti-biofilm strategies.

## Supplementary material

10.1099/jmm.0.002186 Supplementary Material 1.

10.1099/jmm.0.002186 Supplementary Material 2.

## Abbreviations

ADI: arginine deiminase
AMR: antimicrobial resistance
BSI: bloodstream infection
CFS: cell-free supernatant
CV: crystal violet
DEG: differentially expressed gene
HAI: hospital-acquired infection
MRSA: methicillin-resistant *Staphylococcus aureus*
MSSA: methicillin-sensitive *Staphylococcus aureus*
MWCO: molecular weight cut-off
NCBI: National Center for Biotechnology Information
PIA: polysaccharide intercellular adhesion
QS: quorum-sensing
RT: room temperature
TSB: Tryptone Soya Broth
