# Supplementary material for: The metabolic trap: Candida parapsilosis inhibits Staphylococcus aureus biofilm maturation by disrupting pH homeostasis and inducing premature exodus
Source: J Med Microbiol. 2026 Jul 28;75(7):002186. doi: 10.1099/jmm.0.002186 (PMC13421106; doi:10.1099/jmm.0.002186)
Supplement: Supplementary Material 2. [file jmm-75-02186-s002.pdf]

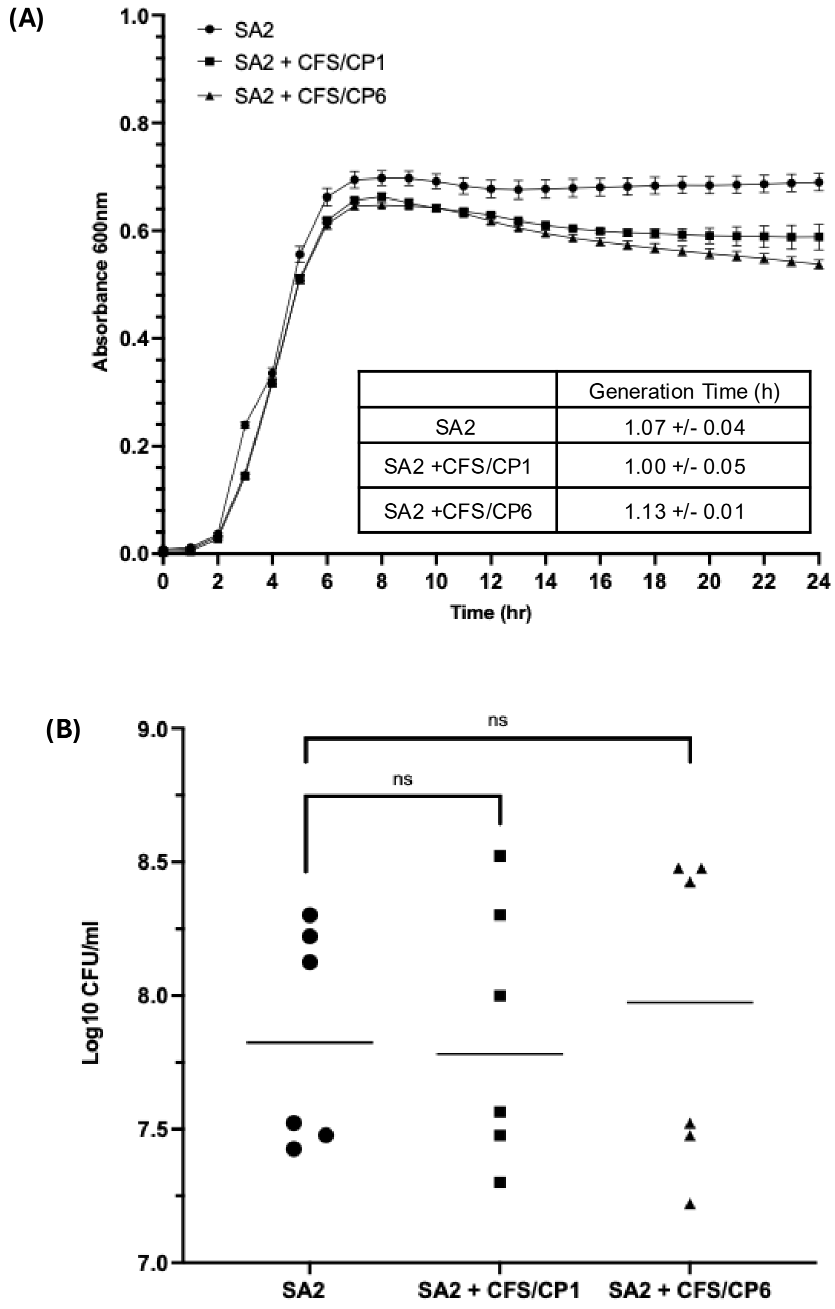

	Generation Time (h)
SA2	1.07 +/- 0.04
SA2 +CFS/CP1	1.00 +/- 0.05
SA2 +CFS/CP6	1.13 +/- 0.01

Fig. S1. Effect of *C. parapsilosis* cell-free supernatant (CFS) on *S. aureus* SA2 planktonic growth kinetics and viability. (A) Planktonic growth curves (OD600) of *S. aureus* SA2 monitored over 24 h in TSB 0.2G supplemented with or without 50% (v/v) CFS/CP1 or CFS/CP6. Inset table displays calculated exponential generation times (h) presented as mean +/- SD. **(B)** Absolute cell viability (log10 CFU/ml) quantified at the 24 h endpoint; horizontal lines represent the group means. Individual data points represent independent biological replicates. Statistical analysis was performed using one-way ANOVA followed by Dunnett's multiple comparisons test (ns, non-significant, $P > 0.05$).
